# Isocyanide-Based Multicomponent Reactions for the Synthesis of Heterocycles

**DOI:** 10.3390/molecules21010019

**Published:** 2015-12-23

**Authors:** András Váradi, Travis C. Palmer, Rebecca Notis Dardashti, Susruta Majumdar

**Affiliations:** Department of Neurology, Memorial Sloan Kettering Cancer Center, New York, NY 10065, USA; varadia@mskcc.org (A.V.); palmertr@grinnell.edu (T.C.P.); rebecca.dardashti@mail.huji.ac.il (R.N.D.)

**Keywords:** Ugi reaction, heterocycles, nitrilium trapping

## Abstract

Multicomponent reactions (MCRs) are extremely popular owing to their facile execution, high atom-efficiency and the high diversity of products. MCRs can be used to access various heterocycles and highly functionalized scaffolds, and thus have been invaluable tools in total synthesis, drug discovery and bioconjugation. Traditional isocyanide-based MCRs utilize an external nucleophile attacking the reactive nitrilium ion, the key intermediate formed in the reaction of the imine and the isocyanide. However, when reactants with multiple nucleophilic groups (bisfunctional reactants) are used in the MCR, the nitrilium intermediate can be trapped by an intramolecular nucleophilic attack to form various heterocycles. The implications of nitrilium trapping along with widely applied conventional isocyanide-based MCRs in drug design are discussed in this review.

## 1. Introduction

Multicomponent reactions are powerful tools for the rapid generation of molecular diversity and complexity. In a typical multicomponent process, more than two components are combined in a single reaction, thereby providing an operationally effective and highly modular approach towards the synthesis of structurally diverse molecular entities. Multicomponent reactions (MCRs) represent an excellent tool for the generation of libraries of small-molecule compounds and are indispensable for structure–activity relationship (SAR) studies. Some MCRs generate uncommon, if not unique, scaffolds so the ability to further functionalize or modify them is key to exploring the utility of the scaffold in the biological realm. The unusual structure of many of these scaffolds makes them suited for exploring biological targets that traditional scaffolds do not target. Novel scaffolds are becoming more and more sought after as pathogens mutate to become resistant to current medications, and anti-aging agents are needed to tackle diseases such as Alzheimer’s, Parkinson’s, diabetes, and cancer. While MCRs may not be directly responsible for the drugs that treat these diseases in the future, they will certainly be involved as scientists search for tomorrow’s remedies. Some of the classic MCRs include the Passerini, Ugi, Mannich, Kabachnik-Fields, Biginelli, Hantzsch, Bucherer-Bergs, van Leusen and Strecker reactions. Isocyanide-based reactions form the backbone of today’s MCR chemistry, and the most common examples are: the Passerini reaction and the Ugi reaction. This review will cover some aspects of isocyanide chemistry with particular emphasis on nitrilium trapping.

### 1.1. Passerini Reaction

**Scheme 1:**
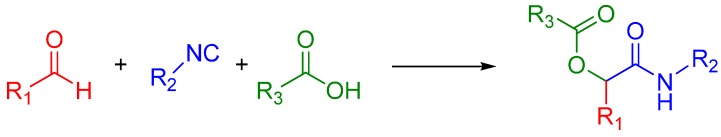
A general Passerini reaction yielding an α-acyloxy amide.

**Scheme 2:**
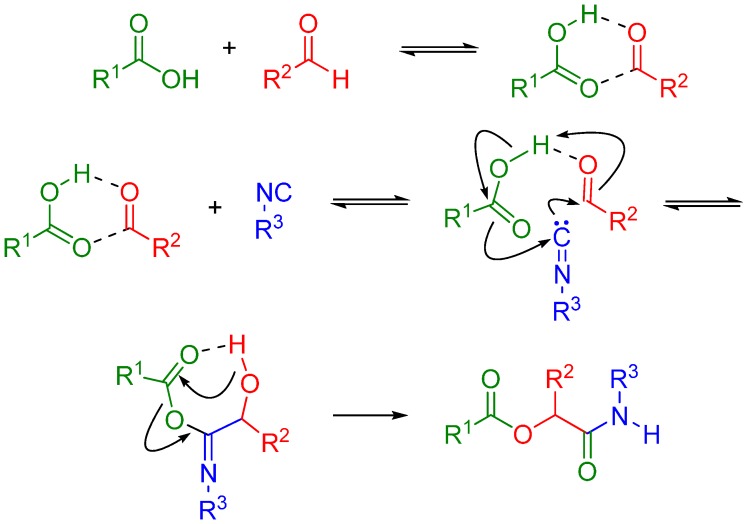
Possible mechanism of the Passerini reaction.

The Passerini reaction is named after Mario Passerini, who discovered this reaction in 1921. It is the first isocyanide based MCR and plays a central role in combinatorial chemistry today. It involves an aldehyde or ketone, an isocyanide, and a carboxylic acid and offers direct access to α-hydroxy carboxamides (Scheme 1). The exact mechanism is a subject of some uncertainty; the mechanism postulated by Ugi suggests a non-ionic pathway, since the reaction is accelerated in aprotic solvents (Scheme 2) [1]. The electrophilic activation of the carbonyl group is followed by a nucleophilic attack by the isocyanide. This creates a nitrilium intermediate, which is then attacked by the carboxylate. 

**Scheme 3:**
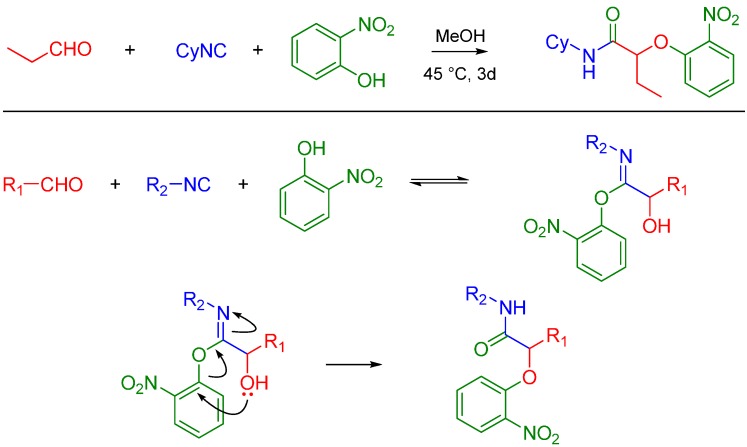
Passerini-Smiles reaction and its possible mechanism.

Asymmetric Passerini reactions are now known using a tridentate indan (pybox) Cu(II) Lewis acid complex developed by Schreiber [2] and other enantioselective catalysts (a combination of silicon tetrachloride and a chiral bisphosphoramide) developed by Denmark [3]. Recently, Wang *et al*. used commercially available chiral Lewis acids to achieve asymmetric reactions [4]. Passerini reactions with alcohols, isocyanides and carboxylic acids have been reported by the Zhu lab, thereby extending the potential utility of this reaction beyond carbonyl containing compounds. The methodology utilizes the conversion of an alcohol to an aldehyde using catalytic TEMPO, CuCl_2_ and NaNO_2_ [5]. In the presence of In(III), Passerini-type reactions have been reported between free alcohols (isopropanol), aldehydes (unsaturated and aryl) and isocyanides such as *t*-butyl isocyanide [6]. Pioneering work by El Kaïm and Grimaud led to the discovery of what is now known as the Passerini–Smiles reaction. In this case, an electron deficient phenol such as 2-nitrophenol (or other nitrogen heteroaromatic but electron deficient phenols) replaces the carboxylic acid. The mechanism is believed to involve activation of the aldehyde by the weakly acidic phenol (pK_a_ ~ 4.2) which makes the carbonyl electrophilic vulnerable to attack by the isocyanide. The incipient nitrilium ion formed is attacked by the phenol followed by an S_N_Ar leading to an α-aryloxy amide. The key step is believed to be the irreversible Smiles rearrangement of the intermediate phenoxyimidate adduct (Scheme 3) [7].

Some other variations of the Passerini reaction with surrogates of carboxylic acid involved in the intramolecular trapping of the nitrilium will be discussed later in the review. The Passerini reaction has recently been used to synthesize a library of monomers for the synthesis of polymers. By varying the R groups in the Passerini reaction, the group was able to tune the properties of the resulting polymer [8]. It also found use in the synthesis of depsipeptides on a dihydropyridinone core [9], dendrimers [10], photocaged compounds [11], cytotoxic/anticancer agents [12,13], imaging agents [14], and HCV NS3 protease inhibitors [15].

**Scheme 4:**
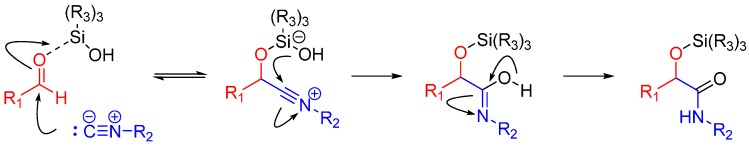
Mechanism of α-siloxyamide-forming Passerini reaction.

A more recent but relevant addition by Soeta *et al.* is use of silanols instead of carboxylic acids in the Passerini Reaction, which allows the synthesis of α-siloxyamides. The mechanism entails coordination of the silyl group to the oxygen of the carbonyl. This renders it susceptible to nucleophilic attack by an isocyanide, followed by intramolecular trapping of the nitrilium ion by the alcoholic functional group of the silanol (Scheme 4) [16].

**Scheme 5:**
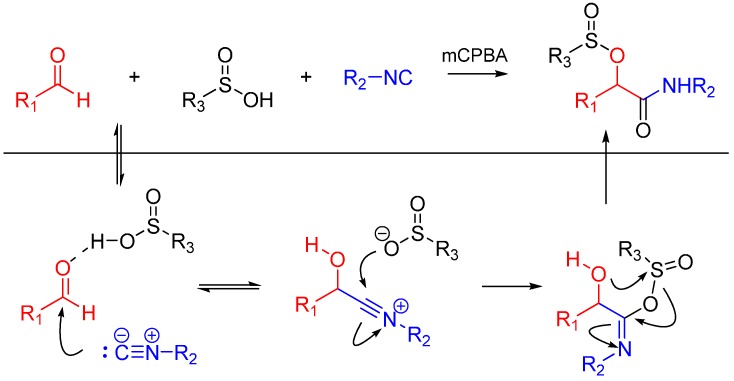
Passerini reaction yielding α-(sulfonyloxy)amides.

Soeta *et al.* also reported the one-pot synthesis of α-(sulfonyloxy)amide derivatives using an oxidative Passerini reaction. An aldehyde, an isocyanide and sulfinic acid were reacted, followed by the addition of meta-chloroperoxybenzoic acid giving the products in high yields (Scheme 5). The reaction has a wide scope on aldehydes and isocyanides [17].

**Scheme 6:**
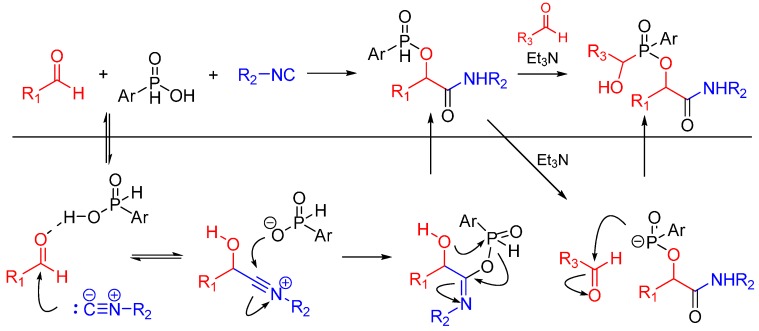
Passerini/Pudovik type reaction leading to highly functionalized α-(phosphinyloxy)amides.

The same group reported a Passerini/Pudovik type reaction between aldehydes, isocyanides and phosphinic acids leading to α-(phosphinyloxy)amide derivatives. This report is the first instance of an isocyanide-based MCR using phosphinic acid instead of a carboxylic acid as a reactant. The nucleophilic phosphinate group undergoes a subsequent catalytic Pudovik-type reaction, affording the products in good yields (Scheme 6) [18].

### 1.2. Ugi Reaction 

**Scheme 7:**
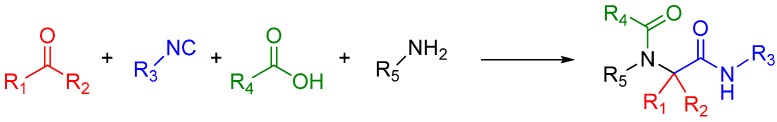
A standard U4CR. The reaction can tolerate a wide variety of R groups.

**Scheme 8:**
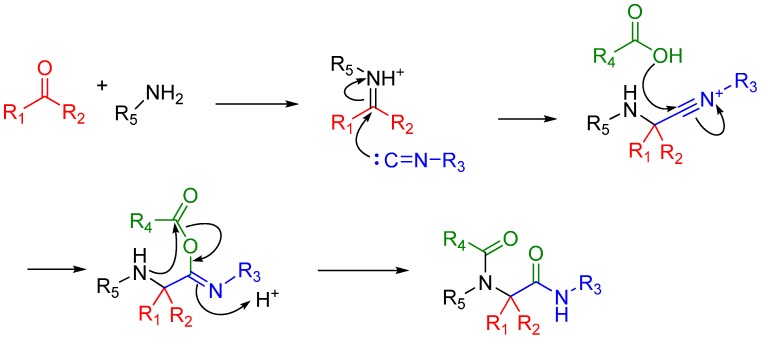
Mechanism of the Ugi 4-component reaction.

A traditional Ugi four component reaction (U4CR, Scheme 7) employs a ketone or aldehyde, a carboxylic acid, an isocyanide, and an amine. The reaction is typically carried out in methanol or 2,2,2-trifluoroethanol in high reactant concentrations. The initial step is the formation of an imine from the amine and the carbonyl compound. This is followed by the nucleophilic attack of the isocyanide, resulting in the formation of the highly reactive nitrilium intermediate. The nitrilium is then attacked by the carboxylic acid, and as a result of intramolecular Mumm rearrangement, the reaction yields a central bis-amide (Scheme 8).

Depending upon the R groups, post Ugi reactions have been reported. Most notable are the Ugi-Heck [19], Ugi-Diels-Alder [20], Ugi-click [21] and Ugi-Buchwald-Hartwig [22] reactions, whereby the Ugi bis-amide with reactive functional groups undergoes secondary reactions to form a ring. Linear bis-amides on the other hand are useful in the synthesis of peptides (linear and cyclic) and peptidomimetics [23].

**Scheme 9:**
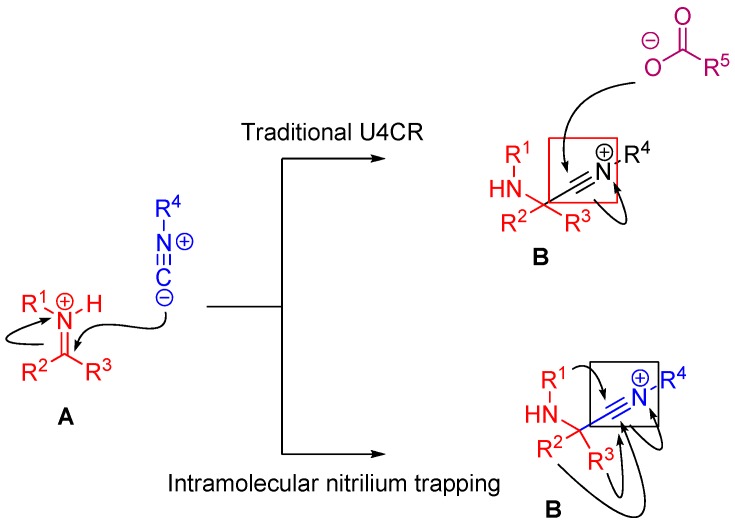
In nitrilium trapping reactions, an external or intramolecular nucleophile attacks the nitrilium species (**B**).

In the case of Ugi and Passerini reactions, an important intermediate in the mechanism is the reactive nitrilium. The mechanism of the Ugi reaction involves the nucleophilic attack of the isocyanide on the imine species (**A**) formed by the condensation of the amine and the ketone or aldehyde (Scheme 9). Lewis acids (such as TiCl_4_) are effective in aiding the formation of the imine by activating the aldehyde or ketone, thereby facilitating a nucleophilic attack by an amine [24]. The resulting nitrilium intermediate (**B**) itself is highly reactive as well and readily reacts with nucleophiles. In a traditional U4CR, the reaction partner of the nitrilium (**B**) is the carboxylic acid. Besides being a nucleophilic partner in the Ugi reaction, the carboxylic acid also plays a part in activating the nitrilium ion. Metal triflates have been shown to activate this nitrilium intermediate making them more susceptible to nucleophilic attack by carboxylic acids and thereby increasing rates of U4CR product formation by about two- to seven-fold [25]. However, when reactants with multiple nucleophilic groups (bisfunctional components) are used in the Ugi reaction, the nitrilium intermediate can be trapped by an intramolecular nucleophilic attack (Scheme 9).

### 1.3. Nitrilium Trapping by an External Nucleophile

**Scheme 10:**
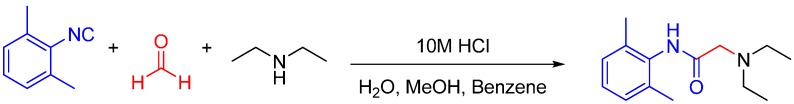
Lidocaine is a local anesthetic on the WHO Model List of Essential Medicines, which can be synthesized in one step via an U3CR.

The traditional Ugi reaction utilizes a carboxylic acid as the fourth component in trapping the nitrilium ion. In the absence of a carboxylic acid, water can act as the nucleophilic partner too. One well-known example of utilizing the Ugi reaction in bioactive molecule synthesis is the synthesis of the local anesthetic lidocaine (Scheme 10) [26]. Trapping of nitrilium ion by water is usually carried out under acidic conditions [27,28]. 

**Scheme 11:**
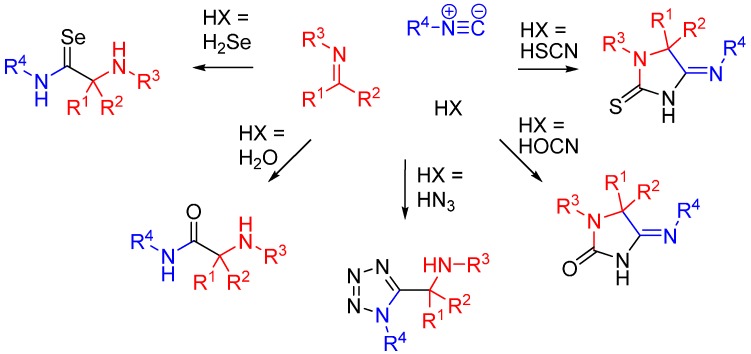
Replacement of the carboxylic acid in an U4CR.

Besides carboxylic acids, hydrazoic acid, HOCN, HSCN, and H_2_Se are some other external nucleophiles which participate as the fourth component in a U4CR (Scheme 11) [29].

**Scheme 12:**
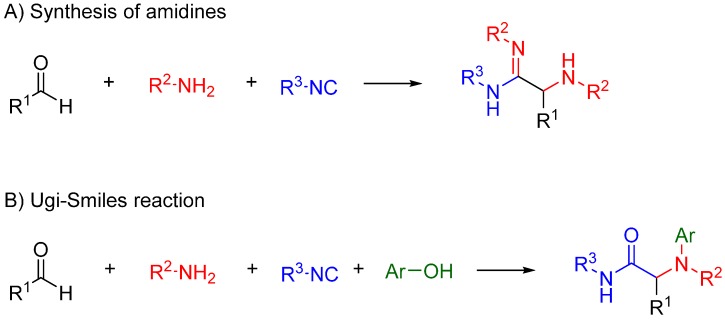
(**A**) Reaction of amines with the nitrilium yields amidines; (**B**) trapping with phenols leads to N-aryl amines (Ugi-Smiles reaction).

Nitrilium trapping by primary amines leads to the synthesis of amidines [30] (Scheme 12A), while with electron deficient phenols leads to *N*-aryl amines [31,32] (Ugi-Smiles coupling, Scheme 12B).

## 2. Intramolecular Trapping of Nitrilium Ion

This review will focus on the chemistry of nitrilium trapping, and its possible application in the synthesis of biologically active heterocyclic compounds. For excellent reviews on the usefulness of MCRs in drug development, see the papers by Dömling and Hulme [26,33,34]. In the absence of an external nucleophile, the reactive nitrilium species can be trapped intramolecularly by a bisfunctional nucleophilic component. This component could be an amine, carboxylic acid, phenol, amide, imine, hydrazide, activated carbon or oxime.

### 2.1. Trapping by a Carboxylic Acid

**Scheme 13:**
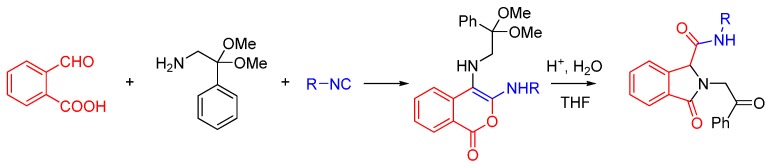
Intramolecular trapping with a carboxylic acid.

In the case where both an aldehyde and a carboxylic acid are present on the same molecule, as in 2-formylbenzoic acid, it has been shown that the carboxylic acid will trap the nitrilium ion [35]. Whereas an U4CR yields a linear scaffold, this trapping generates a cyclic product. This has been utilized for the rapid and high-yielding synthesis of functionalized isocoumarins, such as the anticoagulant warfarin (Scheme 13). Treatment of the product with a catalytic amount of acid led to an isomerization to the expected product.

### 2.2. Trapping by Amide

**Scheme 14:**
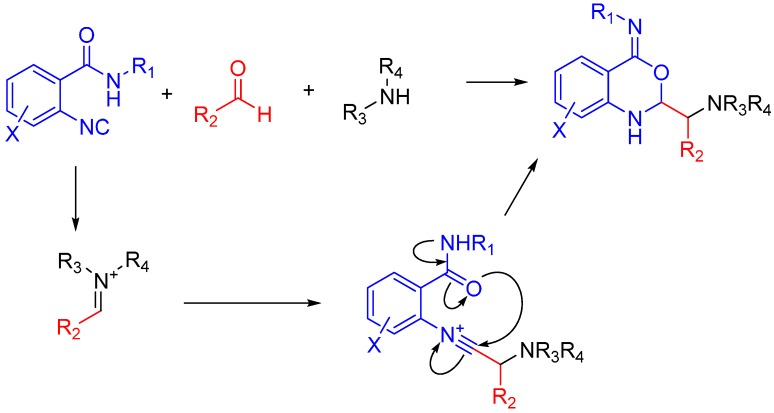
The isocyanide attacks the nitrilium ion but is then trapped by the amide substituent before hydrolysis can occur.

Nitrilium ions can also be trapped by isocyanides containing a secondary amide. The nitrilium is trapped by the amide after attacking the imine, but contrary to what one would expect the nitrogen does not directly attack the isocyanide carbon. Instead, the electrons flow from the nitrogen through the oxygen, and only then to the target atom (Scheme 14). The reaction proceeds even when R^1−4^ are bulky substituents, showing that the scope of the reaction is not limited by the trapping when compared with a traditional Ugi [36].

### 2.3. Trapping by an Imine

**Scheme 15:**
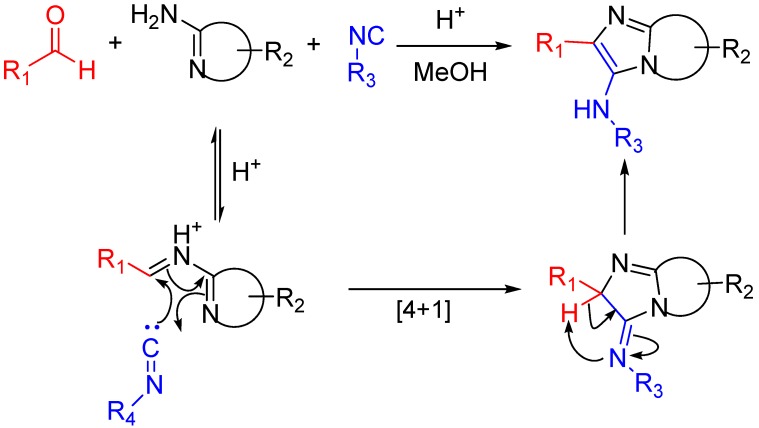
The isocyanide in the Groebke–Blackburn–Bienaymé reaction is trapped by the presence of an imine-like moiety [39].

**Scheme 16:**
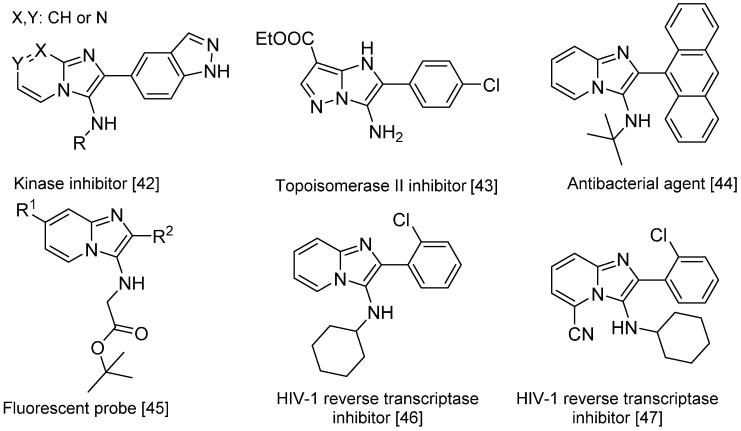
Bioactive compounds synthesized via the Groebke–Blackburn–Bienaymé reaction.

The Groebke-Blackburn-Bienaymé reaction [37,38,39] yields 3-aminoimidizoles in a non-concerted [4 + 1] reaction between an imine formed by the reaction between an aldehyde and an amine, and an isocyanide. The heterocyclic nitrogen traps the nitrilium ion, which leads to the formation of an imidazole ring following a rearrangement (Scheme 15). This reaction is widely used for the generation of highly diverse small molecule libraries [40,41]; the bioactive compounds synthesized this way include kinase inhibitors [42], topoisomerase II inhibitors [43], antibacterials effective against methicillin-resistant *Staphylococcus aureus* [44], fluorescent probes [45] and HIV-1 reverse transcriptase inhibitors (Scheme 16) [46,47].

### 2.4. Trapping by a Secondary Amine

**Scheme 17:**
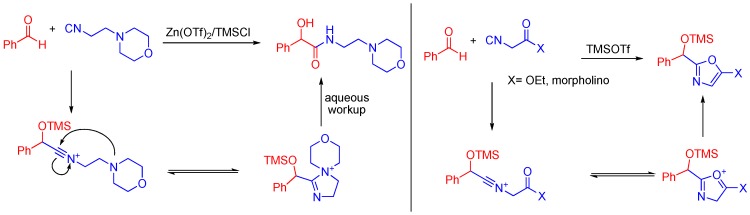
A morpholino isocyanide yields a linear product, while an α-keto morpholino isocyanide cyclizes.

When an aldehyde and 4-morpholinepropionitrile are combined in the presence of trimethylsilyltriflate, the aldehyde is trapped by the TMS group and the morpholino nitrogen stabilizes the nitrilium ion (Scheme 17). The trimethylsilyltriflate coordinates to and activates the aldehyde to attack by an isocyanide, similar to how the carboxylic acid normally activates the aldehyde through hydrogen bonding. If a ketone is present on the α-carbon of the isocyanide, the reaction can yield a cyclized product as well [48].

### 2.5. Trapping by Amine 

**Scheme 18:**
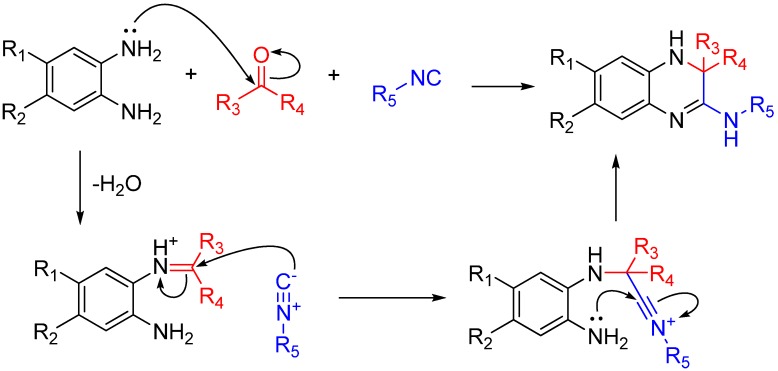
Synthesis of 3,4-dihydroquinoxalin-2-amine and a library of substituted analogs using 1,2-diaminobenzenes.

If the amine reagent is a diamine, one amino group will form an imine and the other will react with the nitrilium after it attacks that imine. This trapping, like many others shown here, forms a highly substituted heterocycle. By using 1,2-diaminobenzenes, the reaction yields quinoxalinones in a single step (Scheme 18). As noted by the authors, several quinoxalinone compounds have affinity for various central nervous system (CNS) receptors, and also may be useful as antidiabetic agents and antiretrovirals [49]. 

**Scheme 19:**
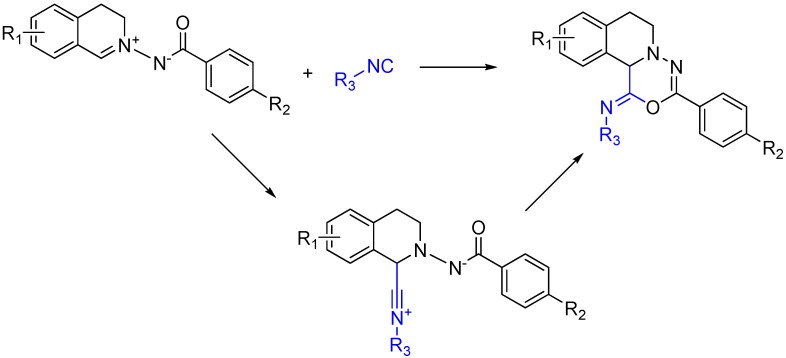
[5 + 1] Isocyanide cycloaddition yields six membered heterocycles.

An intriguing way to ensure the generation of a heterocycle from a trapped MCR is to use a reactant that contains both the isocyanophile, which is an imine in a typical Ugi, and a nucleophile to trap the nitrilium after its attack (Scheme 19). While similar to using a diamino reactant, this approach goes one step further and includes the “imine” with the nucleophile rather than forming it *in situ* [50].

### 2.6. Trapping by Enamide

**Scheme 20:**
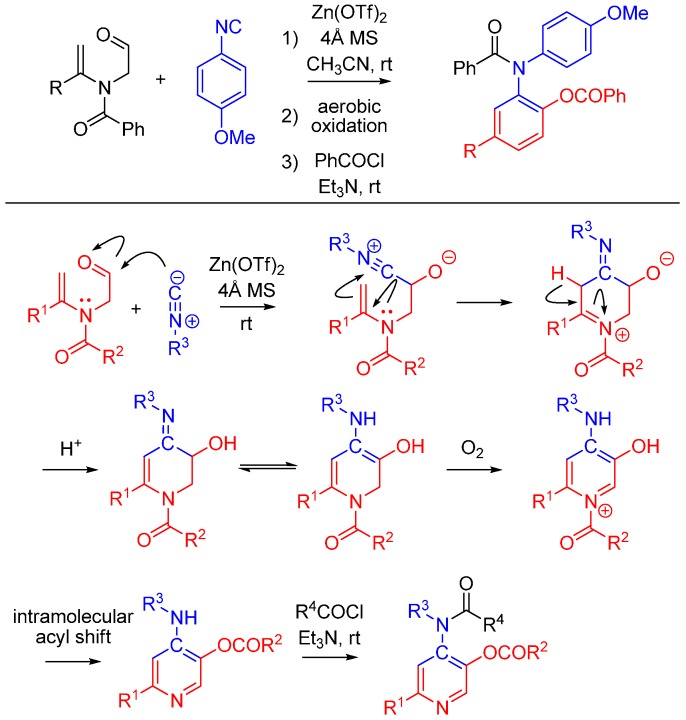
[1 + 5] cycloaddition with isocyanides and enamides yields substituted pyridines.

Lei and co-workers report a novel approach to synthesize pyridines [51]. The method features an unprecedented α-addition of aldehyde and enamide to an isocyanide. While not an Ugi reaction, this cycloaddition is an intriguing example of nitrilium trapping outside the realm of isocyanide-based MCRs. The cascade reaction involves a Zn(OTf)_2_-promoted [1 + 5] cycloaddition of the isocyanide with a substituted enamide, followed by the aerobic oxidative aromatization and intramolecular acyl transfer and finally an acylation by an external acyl chloride to yield 2-substituted 4-acylamino-5-acyloxypyridines (Scheme 20). The method provides a straightforward route to diversely substituted pyridines that are analogues of acetylcholinesterase inhibitors [52].

### 2.7. Trapping by Hydrazide 

**Scheme 21:**
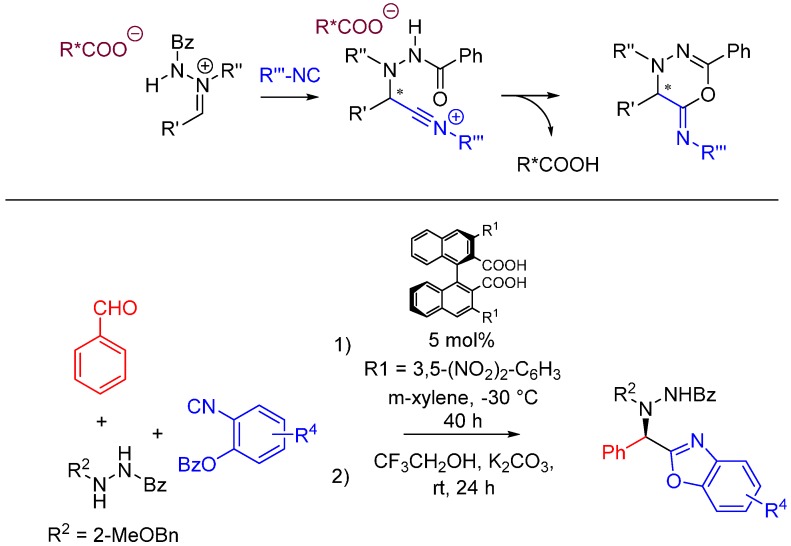
Chiral, acid-catalyzed, asymmetric Ugi reaction.

Achieving stereocontrol is among the current challenges of MCR chemistry. A recent example by Hashimoto *et al.* uses acyclic azomethine imines as prochiral electrophiles generated with catalytic amounts of axially chiral dicarboxylic acids [53]. The nitrilium intermediate following the attack of the isocyanide is trapped intramolecularly by the oxygen of the hydrazide. The imine is generated from benzaldehyde and *N’*-benzylbenzohydrazide in the presence of a chiral carboxylic acid. 2-benzoyloxyphenyl isocyanides [54] are used for this reaction, and a one-pot basic hydrolysis is performed on the Ugi products to generate asymmetric benzoxazoles in high yield and *ee* (Scheme 21).

### 2.8. Trapping by Activated Aryl Carbon 

**Scheme 22:**
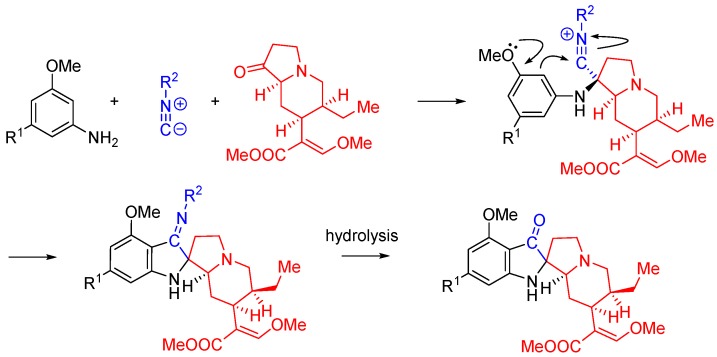
The interrupted Ugi approach to synthesize mitragynine pseudoindoxyl analogs.

Nitrilium trapping can be utilized in the synthesis of natural product analogs as demonstrated by the work of Kim *et al* [55]. In this work, the synthesis of 11-methoxymitragynine pseudoindoxyl, a derivative of the opioid natural product mitragynine, is reported. The electron donating properties of the C-9 phenolic group are utilized to direct the annulation reaction through the intramolecular trapping of the nitrilium species, forming the spirocyclic indoxyl ring system. The Ugi three-component reaction is interrupted by a Houben-Hoesch type cyclization (Scheme 22) [56]. 

**Scheme 23:**
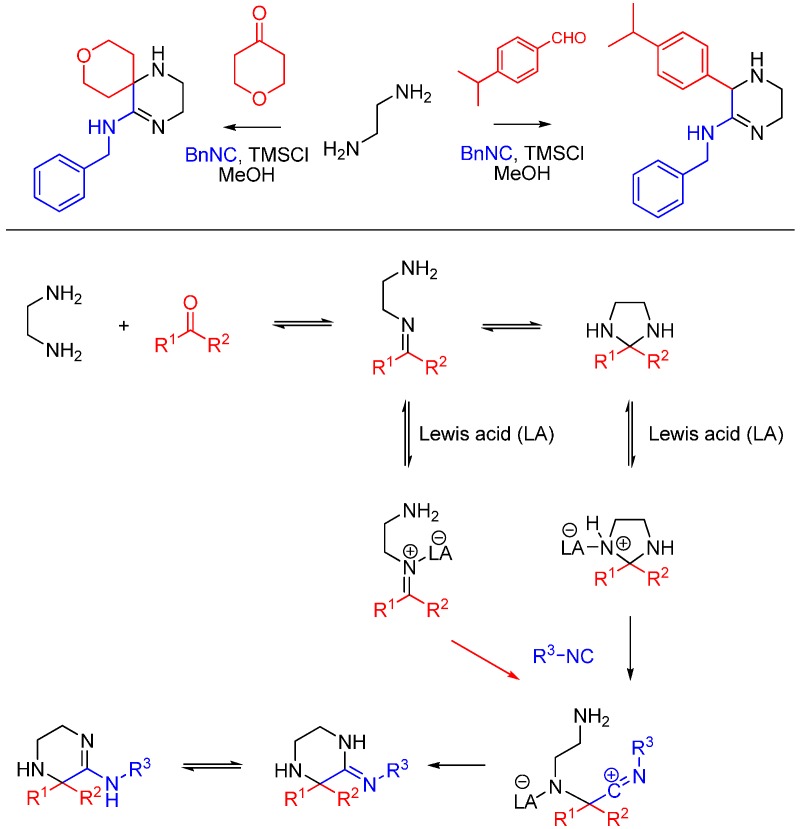
Synthesis of tetrahydropyrazines using ethylenediamine and Lewis acid catalysis.

Kysil *et al.* report a multicomponent reaction between ethylenediamines, isocyanides, ketones and aldehydes that affords highly substituted 3,4,5,6-tetrahydropyrazin-2-amines, including spirocyclic compounds (Scheme 23) [57]. The tetrahydropyrazine ring is formed as a result of the intramolecular trapping of the nitrilium intermediate by the second ethylenediamine amino group and the subsequent tautomerization. The reaction is promoted by Lewis acids, particularly trimethylchlorosilane. 

**Scheme 24:**
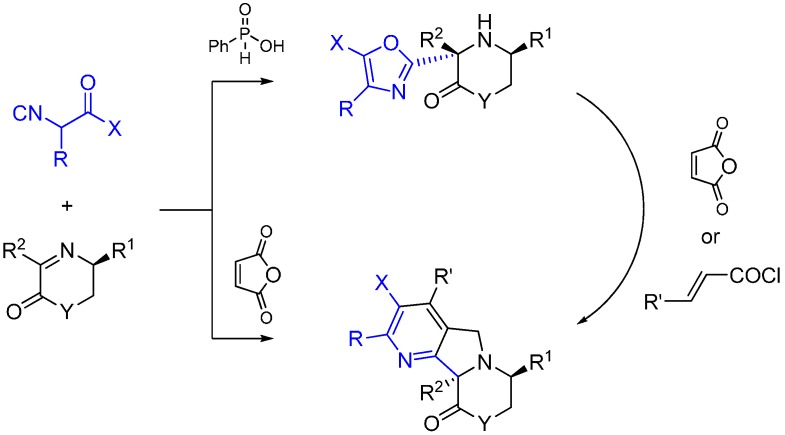
Synthesis of tetrahydropyrazines using ethylenediamine and Lewis acid catalysis.

Xia and co-workers report an asymmetric Ugi-type reaction between α-isocyanoacetamides and chiral imines catalyzed by phenylphosphilic acid leading to 3-oxazolyl morpholin-2-one or piperazin-2-one derivatives [58]. The oxazol ring is formed following the intramolecular attack of the carbonyl oxygen of the isocyanoacetmide amide group. The subsequent reaction of aminooxazoles with maleic anhydride led to fused heterocycles through a multiple domino reaction sequence (Scheme 24).

## 3. Intramolecular Nitrilium Trapping by Aminophenols

Heterocycles play an important role in the design and synthesis of bioactive small molecules: the vast majority of marketed drugs contain at least one heterocyclic ring. Numerous nitrogen-containing heterocycles including dihydrothiazoles, benzoxazoles, benzothiazoles and benzimidazoles possess biological activity [59,60,61,62,63].

**Scheme 25:**
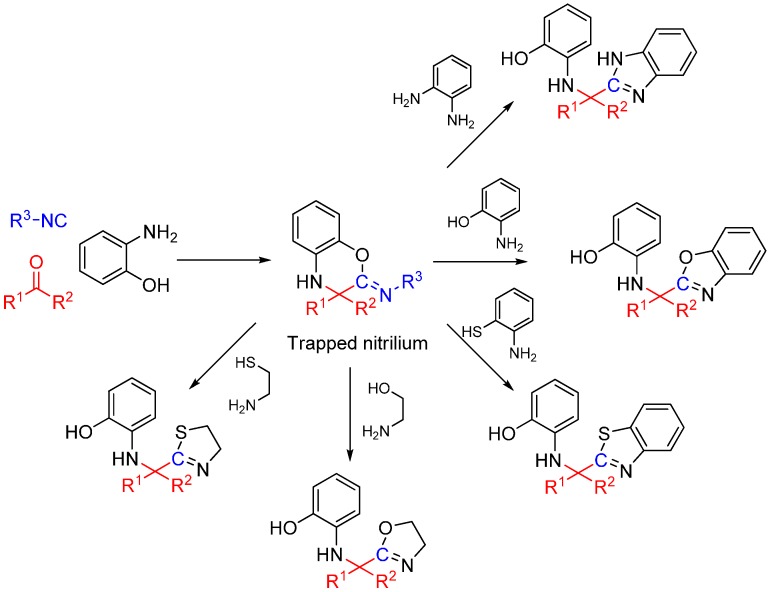
Synthesis of heterocyclic scaffolds using a three-component reaction.

We recently reported a novel MCR between α-ketones, 2-aminophenol, and isocyanide that takes advantage of intramolecular nitrilium trapping yielding benzoxazoles and other heterocycles (Scheme 25) [64]. The reaction proceeds via a benzo[*b*][1,4]oxazine intermediate. Owing to the reactive nature of the trapped intermediate, the ring can be opened by a second molecule of aminophenol or other bis-nucleophiles yielding benzoxazoles and other diverse heterocyclic scaffolds. Furthermore, the reaction generates two additional diversity handles that were utilized to synthesize various substituted bis-heterocyclic derivatives. Synthesis of heterocycles is traditionally accomplished through multistep routes and/or harsh reaction conditions, particularly of benzoxazoles [65], and while MCR approaches can also be taken [29,66], there is a need for a more resource-efficient, preferably one-pot method for their synthesis [67,68]. Metal catalyzed insertion of isocyanides leading to heterocycles are known and usually utilize harsh conditions and have somewhat limited scope in terms of diversity [69,70]. 

**Scheme 26:**
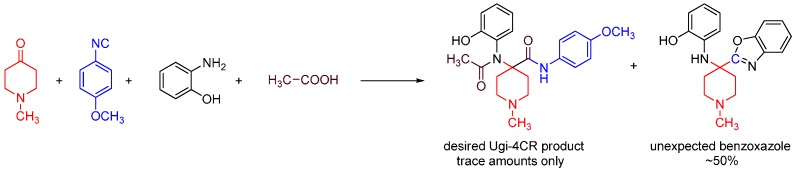
Our MCR yielded an unexpected benzoxazole instead of the U4CR product.

We discovered this reaction while performing a routine U4CR in 2,2,2-trifluoroethanol (TFE) between 2-aminophenol, *N*-methyl-4-piperidone, 4-methoxyphenyl isocyanide and acetic acid at 55 °C, we observed an unusual heterocyclic (benzo[d]oxazol-2-yl)-1-methylpiperidine product (benzoxazole, **1**) along with the expected U4CR product seen only in trace amounts (Scheme 26).

**Scheme 27:**
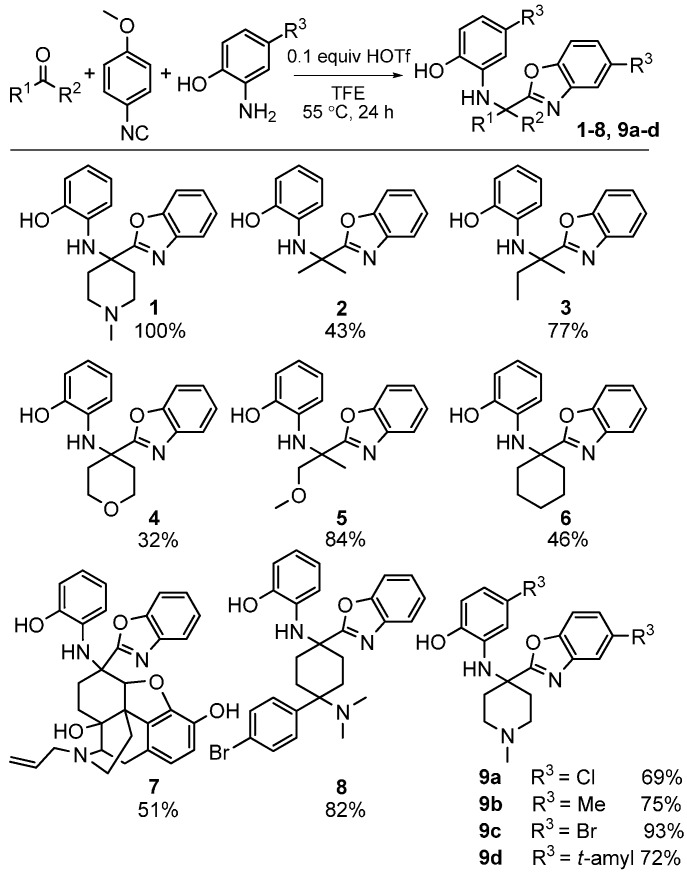
Scope of our MCR yielding benzoxazoles.

We investigated the scope of this heterocycle-forming MCR using various isocyanides. While 4-methoxyphenylisocyanide provided the best yields, other isocyanides were also effective in the reaction. It should be pointed out that the isocyanide contributes only one carbon atom towards benzoxazole; therefore, the reaction outcome is independent of the nature of isocyanide used. The scope of this reaction was evaluated by using different ketones and substituted 2-aminophenols (Scheme 27). The reaction was effective with a variety of ketones including the complex, multifunctional semi-synthetic natural product-like naloxone (product **7**), a clinically used opioid antagonist, highlighting the good functional group tolerance of this reaction.

**Scheme 28:**
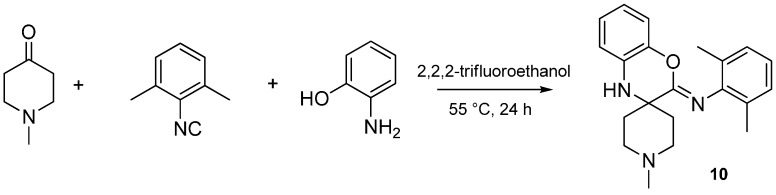
Sterically hindered isocyanide yields a [benzo[*b*][1,4]oxazine]-imine (**10**).

Isocyanides play an unusual role in this MCR, contributing only one carbon atom to the benzoxazole. The aryl or alkyl amine moiety acts as the leaving group, leading to an insertion of only the isocyanide carbon into the benzoxazole moiety. While investigating the effect of various isocyanides, we found that 2,6-dimethylphenyl isocyanide, instead of the expected benzoxazole, yielded a spiro[benzo[*b*][1,4]oxazine]-imine (benzoxazine, **10**) scaffold when reacted with NMP and 2-aminophenol (Scheme 28). The isolation and structure elucidation of this benzoxazine derivative provided us the first clues about the mechanism of this reaction.

**Scheme 29:**
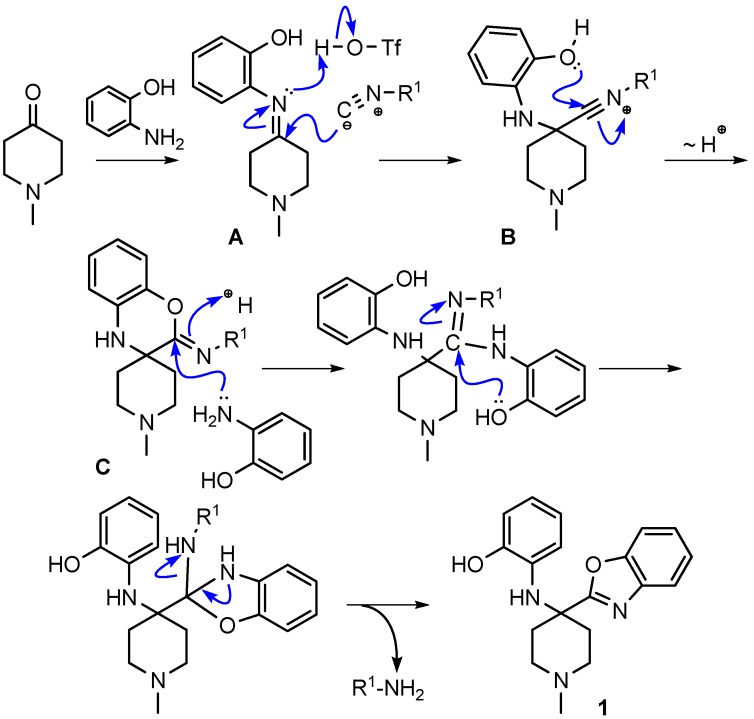
Proposed mechanism for the formation of benzoxazole **1**.

We hypothesized that the benzoxazine **10** was an intermediate in the reaction route leading to the final product benzoxazole **1**. We envisioned the reaction mechanism for the formation of benzoxazoles shown in Scheme 29.

To test our proposed mechanism that the benzoxazine **C** is an intermediate in the synthesis of benzoxazoles, we isolated the benzoxazine obtained using 4-methoxyphenylisocyanide. We enhanced the scope of our MCR by reacting the isolated benzoxazine intermediate with a series of bis-nucleophiles including 1,2-diaminobenzene, 2-aminothiophenol, and cysteamine to synthesize benzimidazole, benzothiazole and dihydrothiazole derivatives, respectively.

**Figure 1:**
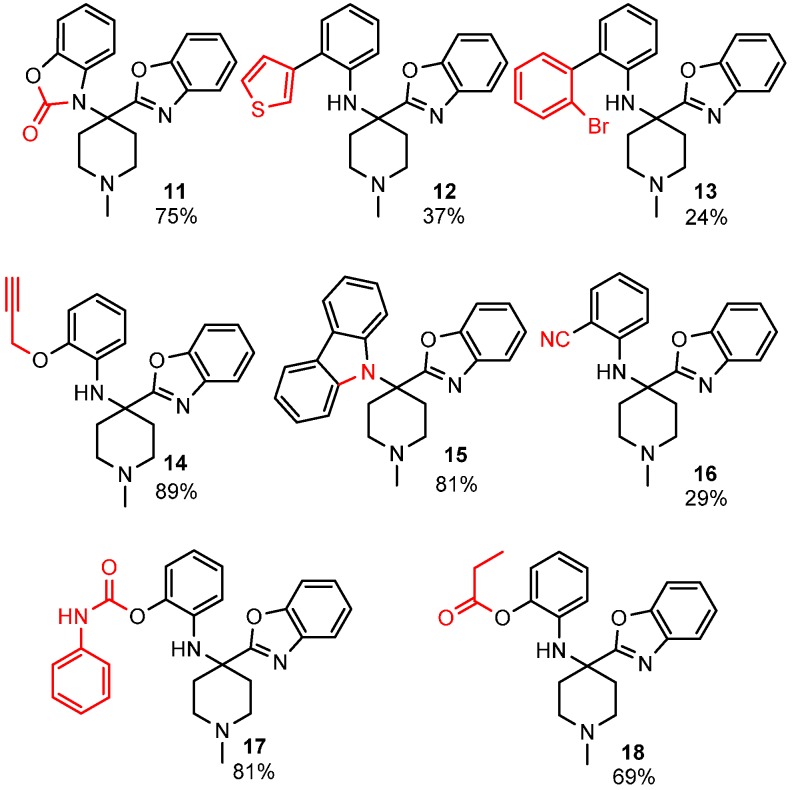
Diversification of the benzoxazole scaffold.

To enhance the diversity and, thus, the possible biological relevance of the benzoxazole scaffold, we decided to exploit the free phenolic OH and secondary aromatic amine of the benzoxazole products to make second-generation derivatives (Figure 1).

In summary, we discovered a convenient, Brønsted acid catalyzed, isocyanide based heterocycle-forming three-component reaction between 2-aminophenols, isocyanides, and ketones that leads to benzoxazoles and other heterocyclic products. The reaction progresses via a benzoxazine intermediate formed by intramolecular nucleophilic trapping of the reactive nitrilium intermediate by an adjacent phenolic group. The reactivity of benzoxazine to bis-nucleophiles was leveraged to synthesize an array of heterocyclic scaffolds including benzimidazoles, dihydrothiazoles and benzothiazoles. The reaction showed good functional group tolerance, and is compatible with different ketones and aminophenols, ideal for the generation of molecule libraries under mild reaction conditions, without the use of transition metal catalysts. We have further exploited the reactivity of the phenolic hydroxyl and the aromatic secondary amine to diversify the scaffolds. 

## 4. Synthesis of Opioids by the Ugi Reaction

Morphine and its semi-synthetic congeners are the most important agents used for the treatment of moderate to severe pain. The desired analgesic effect is accompanied by various dangerous adverse effects, which are predominantly mediated by mu opioid receptors (MOR). One possible way to overcome MOR-mediated adverse effects is to synthesize mixed partial agonists or compounds with mixed MOR agonist/delta opioid receptor (DOR) antagonist properties [71,72]. The ability of morphine to induce tolerance and dependence may be countered by co-administering DOR antagonists without sacrificing antinociception [73,74]. Similarly, partial activation of multiple opioid receptors, for example by a dual MOR-DOR partial agonist ligand, can alleviate these side effects. However, the previously reported MOR/DOR mixed agonists are mostly peptides, therefore their clinical applicability is low [75,76,77].

**Scheme 30:**
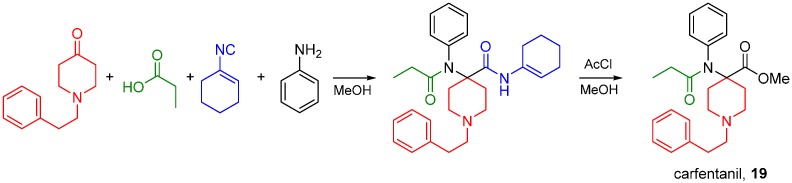
Synthesis of carfentanil by the U4CR.

Synthetic aryl anilido piperidines including fentanyl and carfentanil (19) are potent analgesics acting primarily on MOR, and their scaffold can be accessed by the Ugi reaction. Lofentanyl, structurally closely related to carfentanil, has been shown to possess high affinity for DOR as well [78]. The synthesis of carfentanil was recently reported using an U4CR followed by methanolysis of the carfentanil amide Ugi product (Scheme 30) [79]. The same approach has been used by Portoghese and co-workers to prepare carfentanil-based bivalent ligands for the treatment of opioid withdrawal [80,81]. 

**Scheme 31:**
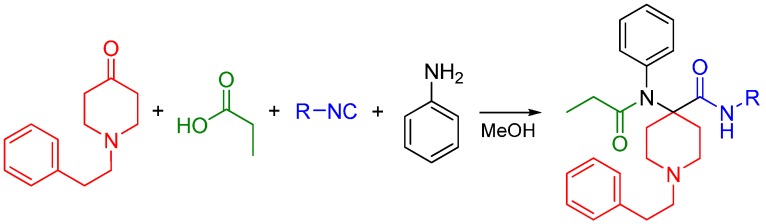
Synthesis of carfentanil amides by the Ugi reaction.

Interested in creating highly potent synthetic opioids acting as MOR and DOR dual agonists, our research group recently reported the synthesis of carfentanil amides using the U4CR approach [82]. We substituted the ester moiety with an amide using various isocyanides and determined if the amide analogs were analgesics with improved side effect profiles compared to morphine. Previously, only the primary amide was reported [83,84]. Four-component Ugi reactions were carried out between *N*-alkylpiperidones, aniline, propionic acid and an array of aliphatic isocyanides (Scheme 31).

Piperidine-4-ones with *N*-phenylethyl and *N*-cyclopropylmethyl substituents were employed in the reactions. The carfentanil amides were isolated in moderate to good yields. The use of Ugi reactions to access the carfentanil scaffold makes diversification library-friendly because of the commercial availability of the simple building blocks used in this reaction.

**Figure 2:**
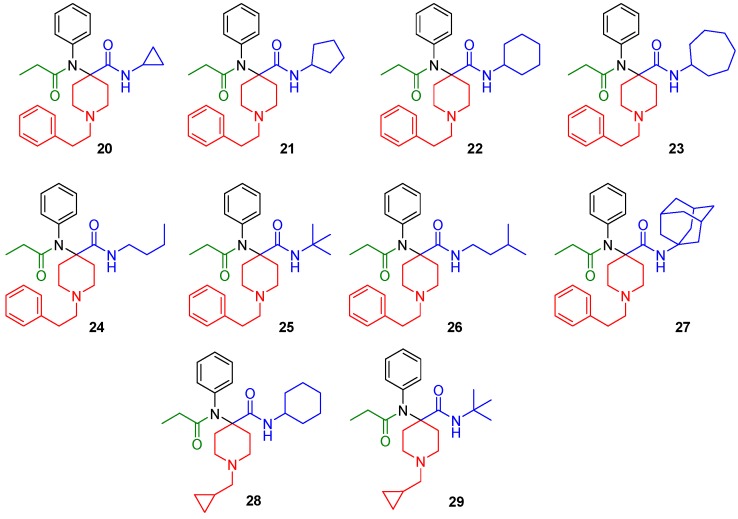
Structures of the synthesized carfentanil amide analogs **20–29**.

The synthesized derivatives (Figure 2, **20**–**29**) were characterized using *in vitro* receptor binding and *in vivo* analgesia assays in a mouse model. Our lead compound, the *N*-cycloheptyl analog (**30**), was subjected to detailed pharmacological characterization.

The synthesized compounds were characterized in *in vitro* radioligand binding assays in CHO cell lines stably transfected with murine MOR, DOR, and KOR (Table 1). Analogs with *N*-cyclopropylmethyl (**28**, **29**) displayed low affinity for opioid receptors (K_i_ > 100 nM), whereas analogs with *N*-phenylethyl substituents (**20**–**27**) showed moderate to high affinity, particularly for MOR. Most analogs had low affinity (K_i_ > 100 nM) for KOR. Three compounds, **22** (cyclohexyl), **23** (cycloheptyl) and **27** (adamantyl) had DOR affinity of less than 10 nM. Given our hypothesis that MOR-DOR compounds may be useful for overcoming side effects, we studied the pharmacology of compounds with affinity at MOR and DOR. We selected **23** for detailed pharmacological evaluation because its affinity for DOR was the highest in the series.

**Table 1:** Summary of *in vitro* receptor binding and *in vivo* tail-flick analgesia.

Cmpd.	MOR-CHO	DOR-CHO	KOR-CHO	ED_50_ (mg/kg)
**20**	10.3 ± 5.1	>100	87.6 ± 29	0.78 ± 0.26
**21**	29.4 ± 15	90.7 ± 23	>100	9.92 ± 0.08
**22**	0.84 ± 0.34	2.65 ± 0.32	0.44 ± 0.05	3.10 ± 0.19
**23**	2.66 ± 1.3	8.90 ± 7.7	>100	10.0 ± 0.00
**24**	21.1 ± 11	87.9 ± 4.8	>100	>10
**25**	2.73 ± 2.2	71.2 ± 8.7	>100	1.09 ± 0.05
**26**	27.0 ± 20	27.0 ± 3.6	>100	>10
**27**	25.0 ± 9.8	8.83 ± 0.63	>100	>10
**28**	>100	>100	>100	>10
**29**	>100	>100	>100	>10

The compounds were evaluated in *in vivo* antinociception assays in mice. Analogs **20**–**23** and **25** showed analgesia at the highest given dose of 10 mg/kg. Three compounds in the series (**20**, **22**, **25**) were more potent than morphine (ED_50_ ~5 mg/kg, sc) [85]. The analgesic ED_50_ values of **21** and our lead compound **23** (ED_50_ = 10 mg/kg, sc) was about two-fold lower than that of morphine (Table 1). Compound **23** was found to be a full agonist at MOR and a partial agonist at DOR in [^35^S]GTPγS binding assays.

We looked at the side-effect profile of **23** in mouse models of respiratory depression (RD) and physical dependence. At doses 4 × ED_50_ (40 mg/kg, sc), **23** did show some signs of RD. Repeated administration of traditional opioids leads to both tolerance and physical dependence, reducing the effectiveness of the drug over time and making it liable to cause addiction. Chronic dosing of **23** produced tolerance, however, **23**-tolerant mice challenged with naloxone (a common opioid antagonist) demonstrated fewer withdrawal symptoms than morphine. Another serious side effect of MOR opioids is constipation. At doses 2× ED_50_ (20 mg/kg) and 5× ED_50_ (50 mg/kg), **23** showed no signs of inhibition of gastrointestinal motility, while morphine caused constipation at its ED_50_ dose. In summary, the cycloheptyl amide analog (**23**) of carfentanil may be useful in negating multiple major side effects seen with classic MOR analgesics. We hope to optimize the structure of this carfentanil amide scaffold to maintain receptor affinities and MOR agonism, while reducing the DOR efficacy to attain a MOR agonist/DOR antagonist based pharmacophore to address respiratory depression, which was observed with the current lead molecule. The utilization of Ugi chemistry to diversify the amine and carboxylic acid ends with commercially available reagents makes further derivatives readily accessible.

The presented examples underline the greatest advantage of multicomponent reactions: the possibility to create diverse bioactive compounds from simple building blocks in just a few or even only one step.

MCRs are an extremely useful and quickly evolving field of synthetic organic chemistry. In recent years, countless varieties of isocyanide-based MCRs have emerged, among which numerous intriguing reactions can be found that take advantage of nitrilium trapping, the nucleophilic attack on the extremely reactive nitrilium intermediate. These reactions have not only introduced us to novel chemical methodologies and reaction mechanisms, but also made new areas of chemical space easily accessible. Based on the diversity of structures that can be accessed by MCRs that involve nitrilium trapping, these methodologies will have a bright future in drug design and synthesis. 
